# Improving electrocoagulation floatation for harvesting microalgae

**DOI:** 10.1016/j.algal.2019.101446

**Published:** 2019-05

**Authors:** Andrew Landels, Tracey A. Beacham, Christopher T. Evans, Giorgia Carnovale, Sofia Raikova, Isobel S. Cole, Paul Goddard, Christopher Chuck, Michael J. Allen

**Affiliations:** a Department of Plant Sciences, Rothamsted Research, Harpenden AL5 2JQ, UK; b Plymouth Marine Laboratory, Prospect Place, Plymouth PL1 3DH, UK; c Interface Analysis Centre, HH Wills Physics Laboratory, University of Bristol, Bristol BS8 1TL, UK; d Centre for Doctoral Training in Sustainable Chemical Technologies, Department of Chemical Engineering, University of Bath, Claverton Down, Bath BA2 7AY, UK; e Amalga Technologies Ltd., 80 Park Road, Hampton Wick, Kingston on Thames, Surrey KT1 4AY, UK; f Department of Chemical Engineering, University of Bath, Claverton Down, Bath BA2 7AY, UK; g College of Life and Environmental Sciences, University of Exeter, Stocker Road, Exeter EX4 4QD, UK

**Keywords:** Electro-coagulation floatation, Microalgae, Separation, Microbubble, Flocculant, High speed atomic force microscopy, Hydrothermal liquefaction

## Abstract

Electro-coagulation floatation (ECF) is a foam-floatation dewatering method that has been shown to be a highly effective, rapid, and scalable separation methodology. In this manuscript, an in-depth analysis of the gas and flocculant levels observed during the process is provided, with microbubbles observed in the 5–80 μm size range at a concentration of 10^2^–10^3^ bubbles mL^−1^. Electrolysis of microalgae culture was then observed, demonstrating both effective separation using aluminium electrodes (nine microalgal species tested, 1–40 μm size range, motile and non-motile, marine and freshwater), and sterilisation of culture through bleaching with inert titanium electrodes. Atomic force microscopy was used to visualise floc formation in the presence and absence of algae, showing nanoscale structures on the magnitude of 40–400 nm and entrapped microalgal cells. Improvements to aid industrial biotechnology processing were investigated: protein-doping was found to improve foam stability without inducing cell lysis, and an oxalate buffer wash regime was found to dissolve the flocculant whilst producing no observable difference in the final algal lipid or pigment profiles, leaving the cells viable at the end of the process. ECF separated microalgal culture had an algal biomass loading of 13% and as such was ideal for direct down-stream processing through hydrothermal liquefaction. High bio-crude yields were achieved, though this was reduced slightly on addition of the Al(OH)_3_ after ECF, with carbon being distributed away to the aqueous and solid residue phases. The amenability and compatibility of ECF to integration with, or replacement of, existing centrifugation and settling processes suggests this process may be of significant interest to the biotechnology industry.

## Highlights

• Electro-coagulation floatation (ECF) can separate or lyse a range of algal species.
• Microbubbles provide lift and the foam can be stabilised by casein doping.
• nm to μm scale analyses show flocculant interactions with undamaged algal cells.
• Oxalate buffer removes contaminating metal flocculants without damaging cells.
• Hydrothermal liquefaction can convert collected biomass to fuel and fertilizer.

## Graphical abstract

**Figure:**
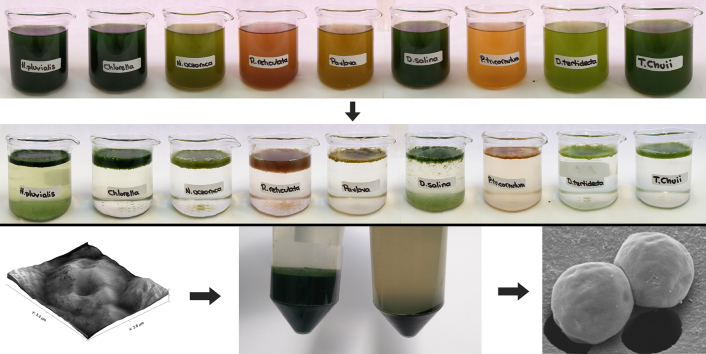

## 1 Introduction

With increasing focus on ‘organic’ and ‘natural’ products, microalgal biotech is poised to increase penetration into the animal feed and nutraceutical markets; which were valued at $31.3 billion and $198.7 billion annually as of 2016 and projected to grow to $34.2 billion and $285.0 billion, respectively, by 2021 [1,2]. The versatility of microalgal biotech has been well documented [3], and the sector is currently experiencing an exponential increase in patents related to microalgae, demonstrating increased potential value in the field [4]. The global microalgal sector was valued at $600 million in 2015, with 91% of the market share held by 3 companies (Solazyme, Algenol, and Sapphire Energy) [5]. Most of this value can be attributed to both increasing microalgal penetration into the oleochemical fatty acid market, and high-value carotenoid astaxanthin production from the microalgae *Haematococcus pluvialis* – the carotenoid market was valued at $369 million in 2014 [6,7]. Whilst whole algal oil production has been valued at $10–13 per gallon [8], there is a need for a more complete biorefinery approach – including separation of high-value products – to make microalgal production economically viable [9]. Testament to this is the failure of Solazyme (initially fuels and chemicals), which became TerraVia (high value oils and whole algae) which went bankrupt in 2017, and is now part of Corbion (Food, nutrient and speciality ingredients).

Whilst many researchers continue to inflate the potential of microalgae by discussing the range of capabilities and applications across the full spectrum of microalgae species [10], there are few developments that add market value in every case. Downstream processing optimisation is one of the few areas of improvement that can be broadly applicable not just to microalgae, but to the biotech sector at large, and has been highlighted as an area in need of development [11], while much R&D continues to focus instead on upstream improvements to algal production via strain selection, growth optimisation and genetic manipulation.

In industrial production of micron-scale algae, removal of excess water (dewatering) is a key step in improving efficiency and concentrating the desired product through volume reduction. Even in the most dense-growing algal species, the organisms only make up around 0.1–1% of the total mass and volume of the entire culture volume [12]. Increasing the cell mass:water ratio by dewatering helps to cut the energy cost of moving liquids and reduces the volume of extraction materials needed in downstream processing. The economics of a number of dewatering methods for microalgae grown in wastewater treatment plants were comprehensively assessed in 1965 by Golueke and Oswald [13], and reviewed 2 decades later [14] showing that gravity-based separation was the most cost-effective technique for lipid production – which still held true when assessed again by Pienkos and Darzins in 2009 [15]. High-throughput centrifugation can be preferable in systems where a long duration between growth and harvest can be detrimental to the quality of final product, offsetting the higher capital expenditure and operating expenditure associated with the technique. Both settling and centrifugation-based processes have been used in wastewater treatment for over a century in some cases, and can be considered mature and completely established in industry; as a result, it is advantageous for any new industrial process intending to make a significant impact to be complementary or readily integrated into existing systems and processes.

Centrifugation, gravity separation, dissolved air floatation and foam floatation are the major methods used for industrial dewatering of algae today, and so augmenting these processes to confer protection to the cells during harvesting, increase separation speed and ease, or reduce energy requirements has the potential to provide improved product quality and/or reduced cost. One such technology is the electro-coagulation floatation (ECF) method, which has been used many times as an alternative for chemical coagulants in wastewater treatment. It is used industrially to treat wastewater containing suspended solids like municipal and household wastewater [16,17], dyes [18], heavy metals like arsenic and cadmium [[19], [20], [21]], oil emulsions such as olive mill wastewater [22], and for removing organisms even of the scale of viral particles when coupled with microfiltration [23], although competing natural organism matter in wastewater can reduce the efficiency of this extraction [24]. Extraction of algae with ECF for biotech applications has also been described multiple times in the literature [16,[25], [26], [27], [28]], however, it has not been used industrially to date to our knowledge. ECF is possible in non-marine conditions; however, due to the much lower conductivity of fresh-water media, it is energetically impractical and results in a large temperature rise which could be detrimental to product quality. It has however been shown that this can be mitigated by adding 1% NaCl (w/v) to the solution without negative effects on the algae [27].

ECF combines the production of metal flocculant and microbubbles in-situ, using a gas-generating cathode and a metal floc-generating sacrificial anode. The two metals commonly used for the sacrificial anode are iron and aluminium, although the comparison between these two, demonstrating the greater flocculating strength of aluminium, has already been reported in the literature and will not be repeated here [[25], [26], [27]]. The chemistry is non-trivial, due to the complex mixture of constituents in the media [25]; however, the main relevant reactions occurring during electrolysis with aluminium electrodes in seawater media are presented in Table 1.

**Table 1:** A list of the main reactions predicted to occur at aluminium electrodes in NaCl solution during electro-coagulation floatation (ECF) treatment. Inert electrodes (such as TiO or carbon) will perform the same set of reactions, excluding any which contain aluminium (Al). As a result, reactions such as **Cl**^−^_(**aq**)_ + **OH**^−^_(**aq**)_ → **ClOH**_(**l**)_ + **2 e**^−^ which produce hypochlorous acid (bleaching agent) occur in a much higher concentration.

Main reactions	Products
Anode	
**Al**_(**s**)_ + **3 OH**^−^_(**aq**)_ → **Al**(**OH**)_3(**aq**)_ + **3 e**^−^	**Al**(**OH**)_3(**aq**)_
**Cl**^−^ + **OH**^−^ → **ClOH**_(**l**)_ + **2 e**^−^	**ClOH**_(**l**)_
**4 OH**^−^ → **2 H**_2_**O**_(**l**)_ + **O**_2(**g**)_ + **4 e**^−^	**O**_2(**g**)_

Cathode	
**2H**^+^_(**aq**)_ + **2 e**^−^ → **H**_2(**g**)_	**H**_2(**g**)_
**Al**^3+^_(**aq**)_ + **3 e**^−^ → **Al**_(**s**)_	**Al**_(**s**)_

Whilst there are numerous side-reactions, the main products that result from this process are Al(OH)_3(aq)_ and H_2(g)_, although some O_2(g)_, ClOH_(l)_ and Al_(s)_ will also be present at lower concentrations. Aluminium hydroxide, or gibbsite as it is referred to in the wastewater industry, acts as a flocculant due to its positive charge in solution at neutral pH, and as mentioned above, has been used in the treatment of a variety of types of industrial wastewater. All suspended particles in water, including gas bubbles, hold a negative surface charge due to their interaction with water [29], and so positively charged ions can act as a glue, forming stable bridges between these suspended particles, resulting in the formation of clumps referred to as flocs. As the flocs form, bubbles suspended in the liquid are captured alongside other suspended particles, such as microalgae. Whilst water has a density of 1 kg per litre, hydrogen gas is a mere 89 mg per litre – approximately 10^5^ times lighter, with other gases having densities lower by a similar magnitude. As a result, any gas volume within a floc can be approximated as having a density close to 0. The implication for this is that a gasless floc will have a density similar to that of the media (albeit slightly heavier, due to the presence of the flocculant), whilst one that contains 20% gas by volume will instead have a density of 0.8 relative to the media. This generates a particle with a large diameter and low density, causing it to rise very rapidly. This is the principal that enables floatation during the ECF process.

The quantity of products of electrolysis can be approximated using Faraday's constant [Eq. (1)], although it is important to note that because of side reactions and loss of gases to the headspace, the approximation will always over-estimate the true amounts present in the system. Ultimately, this enables a rough prediction for the operating conditions suitable for removing a given quantity and species of algae after exponential growth has been determined experimentally; however, due to the complex nature of biological media post exponential growth, it is important to note that this concentration will change from case to case and so experimental verification on the smaller scale is needed for each new system.

In this manuscript, an in-depth analysis of electro-coagulation and electrolysis of microalgal culture is performed. Initially, the levels of flocculant and gas produced during ECF were assessed, with a novel methodology for gas measurement using image analysis described. Culture bleaching with inert electrodes was demonstrated, then the kinetic parameters for ECF separation were calculated. Next, ECF was tested on nine algal species, and high speed atomic force microscopy and light microscopy were used to visually interpret changes to the microalgal cells following ECF treatment. Following these assessments, two improvements were investigated: the addition of spiked protein to improve foam stability and the addition of a wash buffer to remove contaminating metal flocculant. Finally, biomass that was collected with ECF was subjected to hydrothermal liquefaction (HTL) to investigate the effect of ECF on biocrude formation.

## 2 Methods

### 2.1 Strains

Algae strains were obtained from the following culture collections: The Culture Collection of Algae and Protozoa (Scottish Association for Marine Science, Oban, Scotland, UK); *Nannochloropsis oceanica* (CCAP 849/10), *Pavlova* sp. (CCAP 940/3), *Dunaliella salina* (CCAP 19/3), *Tetraselmis chuii* (CCAP 8/6), *Dunaliella tertiolecta* (CCAP 19/24) *Haematococcus pluvialis* (*CCAP 34*/*7*). The Marine Biological Association, Plymouth, UK; *Rhodomonas reticulata* (MBA530). Culture Collection of Algae at the University of Texas, Austin, USA; *Chlorella sorokiniana* (UTEX1230), *Phaeodactylum tricornutum* (UTEX646).

### 2.2 Culture conditions

Stock cultures were maintained under batch culture conditions (4 L in 4.5 L bubble columns) in F/2 medium [30] with a salinity of 33 ppt or Bold's Basal Media, for salt water and freshwater strains respectively, and maintained under 100 μmol photons m^2^ s^−1^ irradiance on a 16 h: 8 h light: dark cycle at 22 °C and sub-cultured on a weekly basis. For scale growth, a fence-style photobioreactor (Bouygues Energies and Services, Manchester, UK) with a working volume of approximately 650 L was used. A photostage consisting of an array of horizontal polycarbonate 50 mm diameter tubes (36 tubes in total, in a 6 tube manifold formation) was illuminated at a light intensity of 450 μmol photons m^−2^·s^−1^, by eight 600 W high pressure sodium lamps. Continuous circulation of the culture via centrifugal pump (up to 30 m^3^ h^−1^) was allowed by stainless steel (316S) pipework connected to a holding tank (200 L). The holding tank was continually sparged with a flux of air (~10 L min^−1^) for oxygen removal, whilst a pH-stat system controlled carbon dioxide delivery. Culture monitoring, which included oxygen saturation, pH, conductivity and temperature measurement, was performed via a Profilux 3 interface. For media composition, F/2 nutrients (Varicon Aqua, United Kingdom) were used in combination with artificial sea water (Instant Ocean, USA) at 35 ppt.

### 2.3 Electro-coagulation flocculation

Except where otherwise stated, all electro-coagulation flocculation experiments were carried out at room temperature in cylindrical vessels, with aluminium electrodes secured in a nylon case. The electrodes were submerged 90 mm in solution (total area 54 cm^2^ per electrode) held in place using a clamp stand, attached to a bespoke 3-phase power supply with a variable voltage fixed at 10 A (Power and Water, Swansea, UK) and stirred with a magnetic stirrer. Flocculation was split into 3 phases: rapid mixing, gentle mixing, and settling. During the rapid mixing phase, the vessel was stirred at 200 RPM whilst electrolysis was induced; during the gentle mixing phase, the electrodes were immediately removed from solution and stirring was reduced to 50 RPM; during the settling stage the stir bar was switched off. Experiments were initially carried out in 2 different vessels: 200 mL in a 250 mL beaker for demonstrating the amenability of different algae to flocculation, and 800 mL in a 1 L beaker for generating biomass for testing effects on the lipid and chlorophyll profiles.

### 2.4 Single-speed mixing

For amenability testing, 200 mL of algal culture was electrolysed at a setting of 10 A for between 5 and 15 s with 200 RPM mixing. The electrodes were submerged to a depth of 2 cm (surface area 12 cm^2^). The electrodes and magnetic stirrer were then both rapidly removed, and the culture was left for a minimum of 30 s to settle. Freshwater species had 2 g NaCl added to them prior to electrolysis.

### 2.5 Biomass growth and experimentation

12 L of culture (PBR grown) was separated in to 1 L aliquots for analysis. Six of these were immediately subjected to ECF for 30 s at 10 A, and then all 12 aliquots were spun at 5000 RPM for 10 min at 4 °C to generate a cell pellet. All pellets were washed in 50 mL distilled water and transferred to 50 mL Falcon tubes, which were then centrifuged for 5 min at 3200 × g at 4 °C, and the supernatant was discarded. Three of the samples subjected to ECF and three of the untreated samples were vortexed with 20 mL of oxalate buffer (0.1 M Na_2_EDTA, 0.2 M MgCl_2_, 0.125 M Tris and 0.125 M oxalic acid, adjusted to pH 6 with NaOH) as described in [31] for 60 s, then left to dissolve the flocs for 15 min. These were then centrifuged for 5 min at 3200 × g, then washed with 50 mL of distilled water 3 times to remove residual flocculant, with the supernatant from each wash stored at 4 °C prior to analysis. The aluminium levels in the supernatant were monitored with the aluminium Palin-test. The biomass from all twelve samples (representing the four treatments in triplicate) was flash-frozen in liquid nitrogen, then freeze-dried overnight. A fraction of the biomass was calcinated in a furnace at 450 °C for 2 h, with dry weights taken prior and post to determine ash content.

### 2.6 Microbubble analysis

10 A was delivered to the experiment for 60 s, then the electrodes and stirrer were switched off and the solution was left to settle for the remaining duration. Oxygen gas was produced at the coated titanium dioxide anode submerged at a depth of 2 cm (surface area 12 cm^2^), whilst hydrogen gas was produced from a circular aluminium foil cathode fixed to the bottom of a 1 L glass beaker (surface area 98 cm^2^) connected by a copper strip through solution, submerged in 800 mL 5 mM citric acid, and the solution was mixed with a magnetic stirrer at 200 RPM. Video was collected on a OnePlus 2 (HDR 2160p@30fps) camera, fixed to a clamp stand and saved in mp4 format. The mp4 was converted to avi format using ffmpeg (downloaded 2018-03-29), audio and colour content was stripped from the file to reduce storage space. The video was converted to a hyper-stack image in Fiji (ImageJ 2.0.0-rc-65/1.51w/Java 1.8.0_66 64 bit) and the average pixel intensity of two rectangular areas, one on the electrode at the top of the image and the other on the magnetic stirrer at the bottom of the image, were collected for each frame. The analysis areas were positioned to avoid large changes in background, focusing primarily on the changes in turbidity of the liquid. The data from this was stored in a csv, and plotting was performed in R using the ggplot2 package [32]. Individual datapoints were plotted with a low alpha value to highlight sparse and dense regions in the data, and overlaid trendlines were generated using a generalised additive model.

### 2.7 High-speed atomic force microscopy (HS-AFM)

The system used in this work was a custom prototype designed by Bristol Nanodynamics which utilised a very low spring constant triangle cantilever (Bruker Nano, MSNL, 0.01–0.03 N/m spring constant) operating in contact mode with a passive mechanical feedback loop. The cantilever vertical deflection was measured by a 2.5 MHz bandwidth laser Doppler vibrometer (Polytec) using a height decoder module. Samples were translated in the fast (1000 Hz) and slow (4 Hz) scan directions by a piezo-actuated flexure stage capable of 3 μm deflection in both axes. Data was collected at 2 million pixels per second and rendered using customized LabView software (2). The high speed flexure stage was mounted on a stick-slip x-y positioner with <100 nm repeatability on both axes (Attocube).

### 2.8 Ash analysis

Aluminium foil squares were weighed on 0.01 mg accuracy balance, 20–50 mg freeze-dried biomass added to foil, loosely folded and weighed again. The initial weight was subtracted from combined weight to determine algal biomass added. The foil was then transferred to solid aluminium weigh-dish, dried for 2 h in an oven at 60 °C to ensure excess water was removed, and weighed again. The weighed dish was then transferred to a muffle furnace at 500 °C for 2 h, and then transferred to a 30 °C drying oven for 30 min to inhibit condensation formation. The vessel was then weighed and subtracted from the pre-muffle weight to determine the final ash content. Weights were determined in triplicate.

### 2.9 Lipid analysis

Fatty acid concentrations and profiles in microalgae cells were determined post conversion to fatty acid methyl esters (FAMEs) using GC–MS (Agilent 7890A GC and 5975C inert MSD, Agilent Technologies Ltd., Edinburgh, UK). Culture samples were centrifuged (10,000 ×*g*), washed in distilled water and resulting pellets lyophilised. To 5–10 mg lyophilised culture, nonadecanoic acid (C19:0) was added as an internal standard and cellular fatty acids were converted directly to FAMEs by adding 1.5 mL of transesterification mix (95:5 v/v 3 N methanolic HCl; 2,2-dimethoxypropane) followed by incubation at 90 °C for 1 h. After cooling, FAMEs were recovered by addition of 1% w/v NaCl solution (1.5 mL) and *n*-hexane (1 mL) followed by vortexing. The upper hexane layer was injected directly onto the GC–MS system as previously described [46]. FAMEs were identified using retention times and qualifier ion response, and quantified using respective target ion responses. All parameters were derived from calibration curves generated from a FAME standard mix (Supelco, Sigma-Aldrich, Gillingham, Dorset, UK). The weight added was multiplied by the non-ash fraction and used to determine the biomass content per sample.

### 2.10 Pigment analysis

2–3 mg of dry algal biomass was resuspended in 360 μL H_2_O with ~300 mg glass beads in 2 mL Eppendorf tubes in quadruplicate. Following disruption for 10 min (Genie2 bead-beater), samples were spun down (20,000 ×*g*) for 10 s to remove bubbles, 1440 μL acetone added, vortexed for a further 2 min, then transferred to the dark and left to equilibrate for 15 min. The tubes were centrifuged for 1 min at 20,000 ×*g* to pellet solid material and the glass beads. 50 μL was pipetted into 150 μL of 80% acetone in a 96 well spectrophotometer plate – again in quadruplicate to determine experimenter error. Absorbance readings were taken at 470 nm, 646 nm, and 663 nm to determine pigment content - as per [33] - briefly C_a_ = 12.21*A663 - 2.81*A646, C_b_ = 20.13*A646 - 5.03*A663, C_t_ = (1000*A470 - 3.27*C_a_ - 104*C_b_)/198, where C_a_ is chlorophyll *a*, C_b_ is chlorophyll *b*, and C_t_ is total carotenoids (μg.mg^−1^).

The weight added was multiplied by the non-ash fraction and used to determine the biomass content per sample. This was then used as a scaling factor to determine the ratio of pigments extracted per gram of combustible mass and multiplied by 36 to correct for dilution (50 μL from 1800 μL). The data was analysed in R, using a paired *t*-test between comparable values to determine significant changes pigment by pigment.

### 2.11 Hydrothermal liquefaction (HTL)

Batch reactors were fabricated according to literature precedent using stainless steel Swagelok® tube fittings [[34], [35], [36]]. The reactor body consisted of a length of stainless steel tubing capped at one end, and connected at the other to a pressure gauge, thermocouple, needle valve, and relief valve. The total internal volume of the reactors was ca. 50 cm^3^. Ash content was assessed by heating 500 mg sample in a Carbolite CWF11 muffle furnace for 5 h (550 °C). Flocculant content was calculated by taking the difference between the ash contents of the flocculated and non-flocculated samples.

Reaction procedures have been reported previously [36]. In a typical reaction, the reactor was loaded with 3 g biomass and 15 cm^3^ freshly deionized water and heated within a vertical tubular furnace set to 700 °C until the specified reaction temperature was reached (345 °C, approx. 11 min), then removed from the furnace and allowed to cool to room temperature. After cooling, gaseous products were released via the needle valve into an inverted, water-filled measuring cylinder to measure gaseous fraction volume. The gas phase is typically composed of 96–98% CO_2_ [36,37]. Hence, gas phase yields were calculated using the ideal gas law, approximating the gas phase as 100% CO_2_. Following this, the aqueous phase was decanted from the reactor contents and filtered through a Fisher qualitative filter paper pre-dried overnight at 60 °C. The product yield in the water phase was determined by leaving a 2.5 g aliquot to dry in a 60 °C oven overnight, and scaling the residue yield to the total aqueous phase mass.

To separate the remaining bio-crude oil and char phase, the reactor was washed repeatedly using chloroform until the solvent ran clear, and filtered through the same filter paper used to separate the aqueous phase (after drying for a minimum of 1 h). The filter paper and collected char were washed thoroughly with chloroform to remove all remaining bio-crude. The filtrate was collected, and solvent removed in vacuo (40 °C, 72 mbar) until no further solvent evaporation was observed visually, and bio-crude samples were left to stand in septum-sealed vials venting to the atmosphere via a needle for a further 12 h to remove residual solvent. The char yield was calculated from the mass of the retentate collected on the filter paper after drying overnight in an oven at 60 °C. Yields were calculated with respect to the overall biomass in the feedstock, excluding flocculant.

## 3 Results and discussion

### 3.1 ECF on salt water

**Fig. 1:**
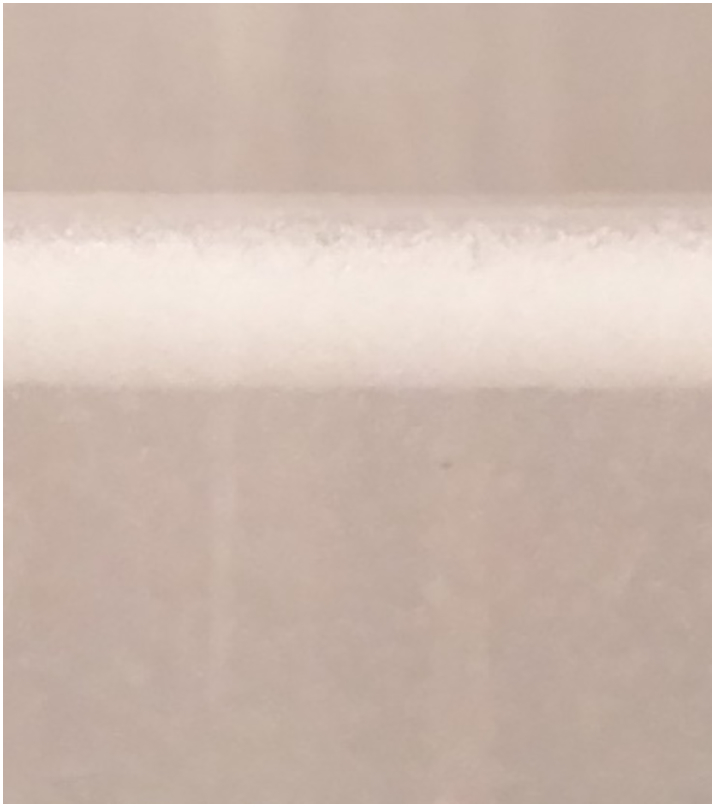
Aluminium hydroxide produced by electro-coagulation floatation (ECF) in blank media forms white hydrogel foam on the surface of the liquid. Particulates in the process of rising through the liquid can be seen in suspension just below the surface, these can sometimes take tens of minutes to eventually settle on the surface or bottom of the flask.

During electro-coagulation floatation (ECF) with aluminium electrodes, aluminium hydroxide (Al(OH)_3_) and hydrogen (H_2_) were initially the major components formed. The Al(OH)_3_ formed a hydrogel in the liquid, binding to any suspended particulates with a negative surface charge from solution and forming a floc. Gas bubbles suspended in liquid (presumably holding a negative surface charge) were also trapped within these flocs, causing their density to be lower than the surrounding liquid, thereby causing their rise to the surface of solution where they formed foam. When the solution treated with ECF contains no coloured particles, such as algal cells, the hydrogel appeared white, as shown in Fig. 1. The longer the process was run, the thicker the foam formed. As the concentration of Al(OH)_3_ increased following excessive application of current, the equilibrium of the chemistry changed, and the hydroxide was converted back into metallic aluminium, causing the hydrogel to become grey in colour.

Using the Faraday constant (Eq. (1)) and assuming complete electrical efficiency, aluminium production by ECF was determined to be 0.269 mg s^−1^A^−1^ Al(OH)_3_, however the dried weights obtained experimentally on water containing NaCl showed variance in the weight of flocculant produced, making accurate experimental values difficult to assess in smaller-scale (200 mL) experiments. Larger scale (1 L) experimental values obtained from media containing algae, show relatively more reproducible concentrations of flocculant (Table 2), suggesting that a combination of volume, time and media-driven side-reactions also affect the chemistry of this process. Biomass subjected to ECF produced a jelly-like pellet following centrifugation, which was drier and easier to collect than the pellets produced from centrifugation alone. The combination of single-pass ECF and centrifugation, compared with single-pass centrifugation (5000*g*, 300 s) alone, enhanced algal recovery from 89 ± 2%, to effectively 100%. To recover lost biomass in the sample not treated with ECF, supernatant and uncollected cells were re-centrifuged and combined with the final replicate of the experiment (Table 2).

(1) $$\mathrm{mass}\;\mathrm{mg}\;s^{-1}A^{-1}=\frac{1000M}{ZF}\eta .$$

**Table 2:** Collected freeze-dried weights of algal samples, with and without electro-coagulation floatation (ECF) treatment. The volume, total duration of applied ECF in seconds-Amps (s A), and the dry weights for the *C. sorokiniana* samples were recorded. All samples were taken from the same batch growth of cells, and so the additional dry weight present in the treated samples results from the ECF treatment. Biomass collection was easier in the ECF treated samples, as the formed pellet was much drier. Leftover biomass, following pellet collection from the untreated samples, was re-suspended in 15 mL of media, pooled with the other 2 replicates, and re-centrifuged in 50 mL volume. For further analysis, the biomass collected from this additional step was pooled into replicate 3, denoted by an *.

Volume (mL)	ECF (s A)	Dried weight (mg)	Average weight (mg)
1000	900	743.83	–
1000	900	770.46	–
1000	900	769.7	761.33
1000	0	459.36	–
1000	0	436.47	–
1000	0	579.71*	491.85

Mass of known electrolysis products produced per ampere per second, where *s* is time in seconds, *A* is amperes, *M* is the molar mass for the molecular components, *Z* is the charge required to produce 1 molecule of product, ℱ is the Faraday Constant, and η is electrical efficiency. η is negatively affected by higher current density (smaller electrode surface area) increasing the overpotential for the reaction.

The experimental values show an average mass increase of 0.299 mg s^−1^ A^−1^ in the ECF-treated samples – higher than the largest possible value assuming complete energy efficiency, 0.269 mg s^−1^ A^−1^ Al(OH)_3_. This increase in mass is likely due to the presence of other suspended particulates and entrapped salts, as well as some lost algae from the untreated method. The centrifuged pellets from the ECF treatment were approximately 10 mL in volume, compared to approximately 5 mL from the untreated pellets. Both pellets were washed 4 times with distilled water, however the ECF-treated pellet was difficult to disrupt and likely retained salts from the growth media. The bulk of this additional volume was media trapped within the metal-flocculant hydrogel, this volume reduced after freeze-drying, resulting in similar final volumes of powder – approximately 2 cm^3^. The media has a predicted dry weight of 10.735 mg mL^−1^ (based on constituents, Table S1), with the post-growth addition of 1% NaCl making up 10 mg of this final mass, and so subtracting this value results in a mass of flocculant between 0.191 and 0.245 mg s^−1^ A^−1^, assuming an even collection of biomass by centrifugation under both conditions and no other unknown solid products arising from ECF, resulting in η = 0.71–0.912. Ash analysis (Table 4) suggests that based on observable flocculant ash, the floc production rate is 0.186 mg s^−1^ A^−1^, or η = 0.69, implying other combustible substances are being captured from the media during ECF. Previous studies suggest that this value may also be an over-estimation. Elemental analysis of the flocs showed that they contained ~40% Al(OH)_3_, with the remaining mass consisting mostly of carbon (~40%), nitrogen (~10%) phosphorus (~7%) and sulphur (~2%) [25]. These extra elements are likely to be a combination of extracellular biological substances in the media: proteins, sugars, polymers like DNA and phosphate from the media can all be captured in the growing flocs, along with waste products generated by electrolysis of the biological constituents of the media.

All samples were freeze-dried for 24 h. The dried pellets from the flocculated cell pellets were more amenable to lyophilisation and subsequent powdering, displaying a dust-like consistency which collapsed to a uniform powder by tapping and shaking by hand. Untreated cultures were close-textured and more difficult to triturate to a uniform powder, requiring a mortar and pestle. Leaving the dried post-flocculated powder in undried air caused it to clump and gel, so all samples were stored in screw-cap sealable brown glass vials at −80 °C to prevent photo-degradation and atmospheric driven rehydration.

### 3.2 Microbubble observations

**Fig. 2:**
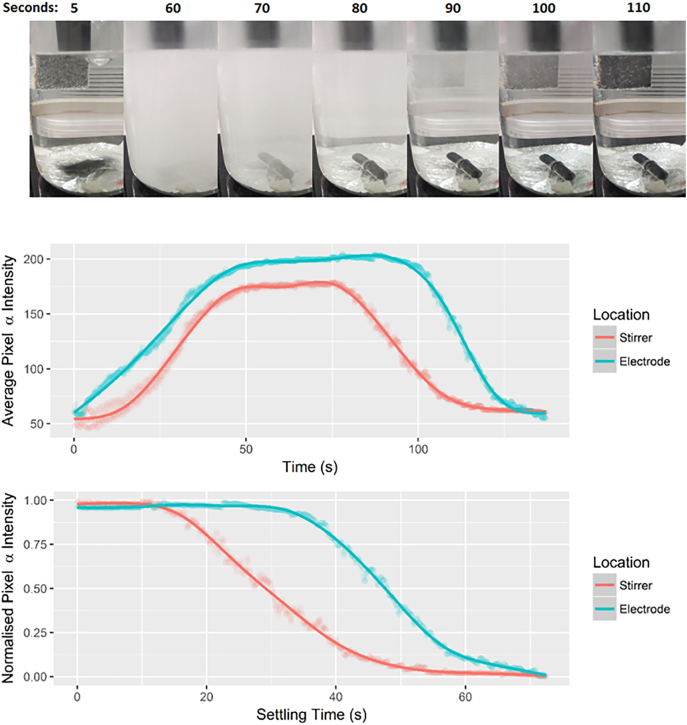
(Top) A time-lapse image of an 800 mL sample of 5 mM citric acid electrolysed with titanium and aluminium electrodes to determine gas content, the cloudiness or turbidity caused by the suspended gas bubbles was analysed against the black parts of the image using densitometry (ImageJ). (Middle) A plot showing turbidity on the stirrer and electrode over the whole experiment. (Bottom) A plot comparing the titanium electrode (top of image) and the magnetic stirrer (bottom of image) following termination of the electrodes and stirring in the experiment. All values were taken from the original video (~28 fps) converted to greyscale, in areas with stable background during the experiment, and the trendlines were generated using a generalised additive model.

To determine the gas residence time without flocculant present, the anode material was changed to titanium oxide and a 5 mM solution of unbuffered citric acid was electrolysed. Using sodium chloride as the charge-carrying agent with titanium electrodes resulted in the production of chlorine gas and hypochlorite, which has potential sterilisation applications, which is discussed further below. In this experimental setup, the area of the cathode was increased to 98 cm^2^ and repositioned to the bottom of the liquid by attaching a circle of aluminium foil to the bottom of the beaker, to accelerate the rate at which bubbles saturated the solution. Hydrogen was produced in solution at an estimated rate of 5.18 cm^3^ s^−1^ A^−1^, oxygen at 2.59 cm^3^ s^−1^ A^−1^. The sample was electrolysed until gas saturation occurred, where the colour of the beaker was homogenous – this occurred after 70 s under these setup conditions – then stirring was halted and clearing time for the liquid was monitored. As can be seen in Fig. 2, the solution had cleared visibly in <60 s after electrolysis, however some micro and nano bubbles that were invisible to the naked eye still remained in solution. These remaining bubbles were visible under green laser light, however this data was qualitative only.

The rate of clearance was measured by densitometry (imageJ) in two locations in the image - the magnetic stirrer (at the bottom), and the electrode (at the top) – both values were normalised to subtract background. As can be seen from the image, the turbidity clears from the bottom more rapidly than the top, demonstrating the updrift in bubbles. This can be seen as a 15–22 second lag between the two datasets in the densitometry (Fig. 2, bottom panel). This lag time is the residence time for the most abundant bubbles in solution to travel the path length of 80 mm, suggesting that they are approximately 80 μm in diameter (as determined by Stoke's settling equation), and the clearance of the majority of the solution in 60 s suggests that the smallest bubbles creating the turbidity effect visible in this experimental setup are not much smaller than 40 μm (Eq. (2)). Volume displacement on the beaker suggested that approximately 20 cm^3^ gas remained in solution in 800 mL when mixing was stopped. This equates to approximately 6 × 10^5^ 40 μm bubbles, or 7.5 × 10^4^ 80 μm bubbles, or approximately 10^2^–10^3^ bubbles per mL. Although not included in this calculation, green laser light identified smaller microbubbles present in solution after separation had apparently completed. This laser-aided microbubble identification could be seen with the human eye but was challenging to capture by imaging, and smaller microbubbles (5–20 μm) were also seen embedded in flocs during microscopy investigation. Bubbles of this magnitude would theoretically take tens of minutes to hours to clear from the solution, and may play a significant role in the floatation of the flocs. As the gas is incorporated into the flocs in the gentle mixing stage, most of the produced gas will be lost before the flocculant concentration is high enough to capture it. This hydrogen gas in the headspace therefore offers an opportunity to recover energy from the process.

(2) $$S_{t}=\frac{d^{2}\left(\rho_{p}-\rho_{l}\right)g}{0.018\mu },d=\sqrt{\frac{0.018\mu S_{t}}{\left(\rho_{p}-\rho_{l}\right)g}}.$$

Stoke's settling equation to determine settling time for spheres in solution; where S_t_ is the settling rate in mm s^−1^, *d* is the diameter of the sphere, ρ_p_ is the density of the particle (for gas this is approximated at 0), ρ_l_ is the density of the liquid (approximated at 1000 kg m^3^), *g* is gravitational acceleration, and μ is the dynamic viscosity of the solution (approximated at 0.001) and the equation was also scaled by 0.001 to convert the units from meters to mm. Next to it is the equation rearranged to give the sphere diameter given a known settling time.

The densitometry also shows that the maximum gradient on the line also changes, with a clearance rate of −0.0327 s^−1^ at the bottom from 20 to 30 s, and −0.0380 s^−1^ at the top from 40 to 50 s. This difference highlights that there is a range of bubble sizes, as if all bubbles were uniform then the rates of clearance would be equal, and therefore there is also a size-fractionation effect occuring during the experimental run. In this case, the more rapid rate of clearing seen at the electrode (top) is likely related to the loss of the larger microbubbles, which produce more turbidity than smaller ones due to larger volumes, therefore producing a more rapid clearing effect. During electrolysis, larger bubbles are gathered in the top part of the solution and are lost more rapidly during the settling stage. This suggests that as the electrolysis is run longer, the proportion of micro and nano bubbles in solution should increase as the larger bubbles are kept at a lower equilibrium due to being lost more rapidly from solution. This effect will be countered by smaller bubbles merging together at a rate related to their relative concentrations, and also the force of down-drift in the solution created by the mixing regime. In this image, it is not possible to disentangle the increased turbidity caused by the larger bubbles from their relative concentration, to determine the relative distribution of different sized microbubbles still in solution.

### 3.3 Sterilisation applications

Using a TiO_2_ anode with salt water produced chlorine gas and hypochlorite (bleach). This same effect was replicated using other innert anodes, such as carbon, and it was noted that since the cathode initially only produces hydrogen gas it can ultimately be made from any conductive material. We tested in-situ bleaching of live culture to determine the potential for utilising salt and an innert electrode as an automated method for sterilising culture in flow. We also performed a qualitative test on combining aluminium and titanium electrodes and flipping the charge on the electrodes during operation to perform flocculation then bleaching, and bleaching then flocculation.

**Fig. 3:**
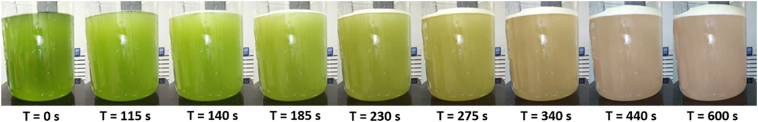
A time-lapse image of an 800 mL sample of *N. oceanica* grown in 33 ppt NaCl media, with titanium and aluminium electrodes, electrolysed at 1 A for 10 min. The current in this experiment was reduced from the normal 10 A, to 1 A, due to safety concerns with high concentrations of chlorine gas being produced. As the cells were lysed, surface foaming was observed in the sample before the colour change. For this culture, the critical level of chlorine to begin lysis was approximately 0.93 mg L^−1^ (140 s), although the more obvious bleaching effects begin at 1.52 mg L^−1^ (230 s). The culture was completely bleached at 2.92 mg L^−1^ (440 s). The thicker foam showed that released protein interacting with the flocculation process successfully captured a greater proportion of released gas from the culture than aluminium hydroxide in salt water (Fig. 1), or algal growth media (Fig. 4, Fig. 5, Fig. 6).

Hypochlorite was created at an estimated rate of 0.533 mg s^−1^ A^−1^. Once hypochlorite was produced, lysing cells began to release denatured protein into the environment, which acted as a foam stabilising agent. This can be seen in Fig. 3, where foam begins to appear on the surface of the culture after the cells begin to break down. Flipping the polarity on the multi-metal electrode setup, where initially the titanium electrode was the anode and then the cathode, first bleached, then flocculated the control cultures. These were then relatively simple to extract by skimming, with increased foam stability and additional gas in solution from the primary bleaching stage. This technique could therefore have potential application in environmental cleanup of harmful algal blooms, where the collected material is then dealt with later by techniques such as hydrothermal liquefaction. It is important to note that the concentration of bleach produced during this process would need to be carefully controlled in an environmental setting, and the technique may ultimately be better suited as a stage of treatment of wastewater or for sterilising ship ballast water, where the hypochlorite can be contained, and the concentration reduced to safe levels with aeration overnight or through the addition of thiosulfate.

Flipping the polarity where the initial anode was the aluminium electrode resulted in incomplete bleaching of the culture, with coloured biomass present in the foam at the surface. Algae embedded within the floc hydrogel were found to be less susceptible to the bleach in solution, although if left undisturbed overnight the culture did still bleach. This demonstrated that once formed, the hydrogel flocs surrounding the algae are not readily disrupted by rapid mixing, even if it causes the gas to be released and inhibits floatation. This gel appears to form a protective layer around the encapsulated cells, inhibiting exchange of materials with the environment, demonstrating potential for the protection of cells and reducing product loss.

### 3.4 Optimised ECF separation

**Fig. 4:**
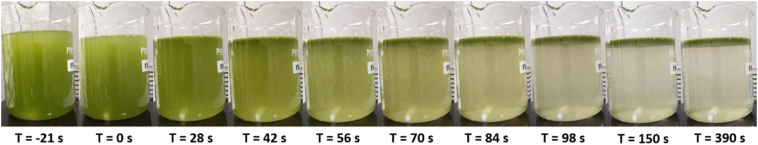
A time-lapse image of an 800 mL sample of *C. sorokiniana* treated with electro-coagulation floatation (ECF). *T* = 0 s was the point where the current and stirring was stopped, the electrodes were removed, and the sample was left to settle. Very little visual difference occurs in the first 30 s, and then a visible clumping event occurred that rapidly cleared the liquid and formed suspended flocs. These flocs grew, collected more suspended gases, and rose to the surface, eventually clearing after 150–390 s, although close observation revealed particles still suspended in solution below the meniscus.

Most previous studies investigating ECF of microalgae kept the conditions constant (ie. continuous electrical current, continuous mixing), however this is not how flocculation is performed industrially, where there is a rapid mixing stage when the flocculant is added, followed by a gentle mixing stage to encourage floc growth, and finally a settling stage. The method was modified to stop rapid mixing after dosing, which allowed the culture to settle and achieved a much more rapid rate of separation, with lower requisite doses of aluminium and overall energy needed for the process. Fig. 4 shows the progression of the flocculation progress after ECF, showing separation with only 2 stages, rapid mixing at 250 RPM during ECF (first pane), followed by settling (remaining panes). Continuing slow mixing at 60 RPM after *T* = 0 reduces the duration of both the lag between the ECF stage and the visible flocculation event and the terminal separation point from 42–84 s and 390 s, to 30–50 s and 180 s, respectively (Fig. 4). For the remaining experiments in this study that were conducted at the >1 L scale, gentle mixing and a 3-minute settling time were used as standard, as there was a negligible difference observed between 150 s and 390 s.

The measured settling times varied from sample to sample, with the exact timings dependent on external factors, including the initial culture density, the duration of ECF, the shape and position of the electrodes in the solution, and the mixing vessel geometry, where dead-zones in non-rounded containers significantly hampered mixing and floc growth. This suggests that determining the separation speed is a combination of a chemical and flow-dynamics problem, accelerated by increasing collision energy between the chemical substrates, but reduced by shear forces in the fluid flow that disrupt growing flocs and release entrapped gas. If the flocculated material is left undisturbed on the surface of the vessel for a period of 4–16 h, the foam ripens and, as gas is released, eventually the flocs break apart and drop from the surface of the liquid to the bottom of the vessel. The stability of the floc remaining on the surface was linked with the duration of ECF, the density of the cell culture, and the age of the cell culture. Ultimately, these factors all contributed to altering the media constituents through the introduction of surfactant molecules, such as proteins or disrupted cells, which in turn stabilised the foam.

The floc-collapse effect can also be achieved by physical disruption of the foam, either with the magnetic stirrer or through interaction with a glass rod or pipette. In addition, running the ECF process to a point where the floc/foam makes up >50% of the volume of the vessel causes some of the floc to settle to the bottom of the vessel, whilst the remainder binds to the surface as foam. This split settling state further exemplifies that most gas is lost during ECF treatment under these conditions, and only interacts with the foam during the floc growth stages where the applied shear is reduced below the formation energy of the floc. Once the ECF treatment produces more hydrogel than can be stabilised by the suspended gas in solution (20 cm^3^ in 800 mL), the remaining gel settles to the bottom of the vessel instead of rising as foam.

**Fig. 5:**
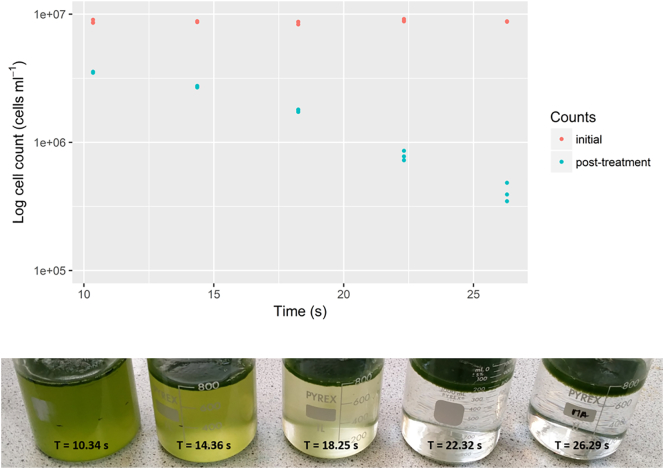
Investigation into electro-coagulation floatation (ECF) treatment duration and separation efficiency of *C. sorokiniana* at 8.7 × 10^6^ cells mL^−1^ (OD_680_ 0.393, OD_750_ 0.266). 800 mL samples taken from the same initial 4 L growth culture were treated with ECF for a range of times between 10 and 30 s. (Top) Cell counts were taken by optical density before (red) and after (blue) ECF treatment. (Bottom) A photograph was taken of all the different ECF time-treatments, demonstrating the relative level of clearance attained over the experiment. The subnatant was collected at a depth of 2 cm below the surface by careful pipetting after 3 min of settling. The thickness of the foam layer increased as a function of ECF duration. (For interpretation of the references to colour in this figure legend, the reader is referred to the web version of this article.)

The initial density of the cell culture is strongly linked to the required length of ECF required to successfully separate cells from solution in the most efficient state possible. Fig. 5 shows a time-series of ECF treatment on a single batch of culture grown in a 4 L bubble column, from an initial cell density of 8.7 × 10^6^ cells mL^−1^ (OD_680_ 0.393, OD_750_ 0.266), which was treated with ECF for between 10 and 30 s. Several features arise from scrutinizing this data: the first is that the rate of flocculation is non-linear. The sigmoidal separation curve has previously been described as having an inflection point around the state where the cells began to be disrupted [26], however, cell disruption is not the major contributing factor in separation under these parameters – instead, it is collision and floc growth. Despite the cultures appearing clear in Fig. 5, the spectrophotometric data still shows turbidity present in solution – this residual absorbance is likely caused by floc-bound particulates and cells that are held at equal or very close to equal density with the liquid and are at their highest concentration just below the foam surface. An example of these can be seen in Fig. 1. Blank media subjected to ECF that had no cells grown in it did not demonstrate this same level of absorbance in the measured wavelengths, presumably as it contained fewer particulates and a higher concentration of flocculant, resulting in larger particulates that settled faster, and so correcting for this effect remains a challenge.

### 3.5 Testing ECF on multiple algal species

**Fig. 6:**
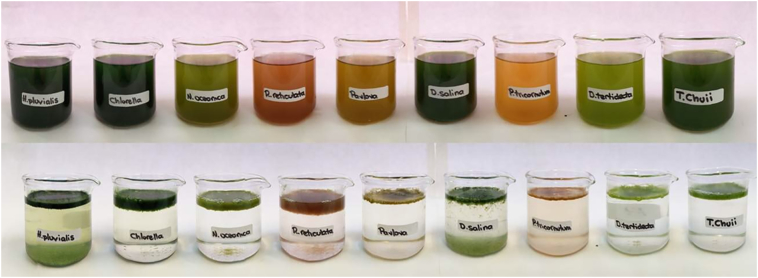
Qualitative before and after images of 200 mL flasks of algal culture treated with electro-coagulation floatation (ECF) for between 5 and 15 s (Left to right: *Haematococcus pluvialis*, *Chlorella sorokiniana*, *Nannochloropsis oceanica*, *Rhodomonas reticulata*, *Pavlova lutheri*, *Dunaliella salina*, *Phaeodactylum tricornutum*, *Dunaliella tertiolecta*, *Tetraselmis chuii*). Several factors affect the smoothness and depth of the flocculated algal foam layer and the clarity of the media presented here, discussed in the text.

A collection of nine different algal species were tested for separation with ECF, to demonstrate that due to the chemical and physical processes involved, the technique successfully separates all different algal species tested (Fig. 6). ECF was found to work in a uniform manner across a variety of different algae displaying different properties (Table 3) although the exact duration required for separation varied based on several factors.

**Table 3:** Different species treated with electro-coagulation floatation (ECF) in Fig. 6, with their sizes, shape, motility and the media the cells were grown in. All strains sizes were obtained from a scanning electron microscopy (SEM) image library of Plymouth Marine Laboratory algal strains, taken by Steve Gschmeissner, PhotoQuest.

Species	Media	Shape	Flagella	Salt tolerant	Size (μm)
*Haematococcus pluvialis*	BBM	Spherical	2	n	20–40
*Chlorella sorokiniana*	BBM	Spherical	0	n	2–10
*Nannochloropsis oceanica*	F/2	Spherical	0	y	2–3
*Rhodomonas reticulata*	F/2	Rod	2	y	3–12
*Pavlova lutheri*	F/2	Spherical	2	y	3–6
*Dunaliella salina*	F/2	Ovoid	2	y	5–8
*Phaeodactylum tricornutum*	F/2	Canoe	0	y	3–30
*Dunaliella tertiolecta*	F/2	Ovoid	2	y	4–7
*Tetraselmis chuii*	F/2	Ovoid	4	y	7–15

The algal cultures were treated in sequence from left to right in the image, and so the images had different times since treatment, variation in culture density, cell size, media, presence of salt, and natural motility was different in each of the different cultures. In addition, these cultures were each 200 mL in volume, causing the rate of concentration increase in solution to be much larger than the 6 L, 1 L and 800 mL volumes that were used to fine-tune the parameters elsewhere in this study. Furthermore, the small volume resulted in a much quicker separation rate, making removing the electrodes and stirrer more time-pressured. This large variance in culture and media properties meant that different lengths of ECF were required for the different samples to generate complete separation, which is responsible for the large variance in foam depth present in Fig. 6, as well as the incomplete separation visible in some samples, notably in *H. pluvialis*.

The foam formed from this protocol is incredibly fragile, suggesting that the gas is not tightly bound within the foam. Accidental disturbance of the foam during picture setup caused some of the floc to fall out of the foam and settle on the bottom, this can be seen most clearly in the *D. salina* and *H. pluvialis* samples. This effect also suggests that the rate of Ostwald ripening within the foam generated by the ECF process is very high. It is also important to note that under these conditions, once treated with ECF for floatation, it was observed that samples cannot be re-treated with ECF for floatation effectively following gas-loss, suggesting that the integration of gas bubbles into the forming flocs, rather than just adherence of bubbles to the edges of the formed flocs, is an important part of the floatation process.

Determining the required dosage of a flocculant for separating different species and conditions is challenging, as flocculated cells are difficult to measure either by counting or absorbance, and the effect is dynamic and cannot be easily halted once it begins to allow accurate measurements in time. As a result, measuring the clarified media in the subnatant after the terminal separation point spectrophotometrically – where the flocculant is saturated in the media and the separation is complete – was used to determine the efficiency. Changes in flocculation properties are classically determined by using an observational technique called a jar test, where observations of changing floc properties and total settling times through a column are recorded, as shown in Fig. 4. The ECF technique introduces additional uncertainty related to the concentration of Al(OH)_3_ from the process; which is affected by the electrode surface, the chemistry of the solution over time, and the suspended particulate density. As can be seen in Fig. 5, the separation efficiency is a function of the concentration of flocculant – or more specifically in this case the duration of ECF – which in turn is directly related the total concentration of cells.

### 3.6 Improving foam stability

**Fig. 7:**
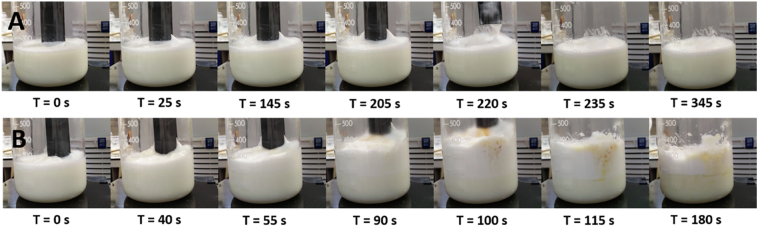
Casein from powdered milk was electrolysed with titanium electrodes at 1 A (A) and 10 A (B). At 1 A, a foam was created that formed soft peaks, however the volume did reduce when the power stopped. The same effect was seen at 10 A, but the volume changes were much more pronounced and rapid, and the large temperature increase (68 °C in 90 s) caused a discolouration of the solution as the sugars burned.

Foam stability is determined by its rate of Ostwald ripening – the rate at which gas can escape from being entrapped within the liquid. The proportion of bubbles stabilised within floc foam can be increased by doping it with a surfactant, such as protein. During the bleaching experiment, a stable foam was produced after cell lysis. To investigate this potentially protein-driven effect, casein in the form of powdered milk was spiked in at 0.1 g L^−1^, equivalent to a slight excess of all cells in a typical algal culture at maximum growth density being lysed. Caesin-stabilised foams are widely used in the food processing industry for the creation of a variety of products, including artisan coffee and ice cream. Electrolysis with titanium electrodes was performed, producing only gas and exposing the solution to electrical current (Fig. 7). This showed that as expected, a stable foam will form, although it collapses to ~50% height rapidly.

**Fig. 8:**
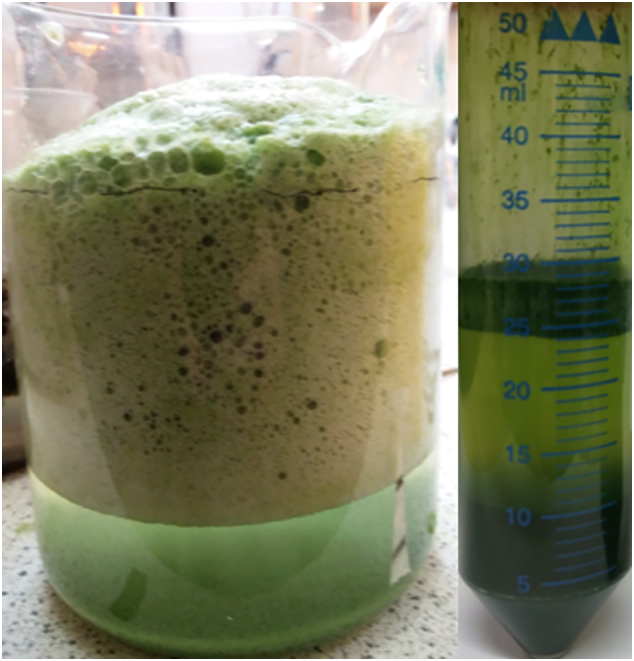
Casein from powdered milk was doped into a culture of *C. sorokiniana*, and the culture was electro-coagulation floatation (ECF) treated. An example of the resulting foam (left) shows that even large bubbles are trapped within solution. The resulting foam from a 6 L culture was collected into 50 mL Falcon tubes and analysed by centrifugation to determine the foam constituents – an example of the output of this experiment is shown (in this figure, RHS). In this case, even after 5 min of centrifugation at 5000 *g*, some of the cell pellet stays bound to the trapped gas and doesn't settle to the bottom of the tube – equivalent to being left to settle for >17 days.

Quantitative measurements from this experiment were difficult to determine; the foam did not sit evenly in solution and disturbing the foam to collect it resulted in loss of gas and therefore volume, so measuring the height or volume was an estimation by eye and a measurement from the liquid to the top of the foam line. Despite the collapse as gas escaped, the milk foam was still substantially more voluminous and stable than the foam produced by aluminium EFC. Purified Cytochrome C was also tested, and similarly produced a stable foam in solution, although under titanium electrodes in the presence of NaCl, its red colour initially bleached before the foam formed. Changing the electrodes to aluminium caused a more stable foam to form than observed with either protein addition or the aluminium hydroxide floc in isolation. The effects of this combined treatment with algal culture are shown in Fig. 8, where after casein doping the foam was stable enough to be collected and measured by centrifugation to determine gas, liquid and solid proportions by volume. The sample of stable foam analysed by centrifugation contained 50% gas (±8%), 35% (±5%) liquid, 15% (±2%) pellet-bound flocculant – an example measurement is shown in Fig. 8. The foam formed from this process is highly explosive, being mostly H_2_ with some residual O_2_ present.

### 3.7 HS-AFM and microscopy of ECF-treated microalgae

**Fig. 9:**
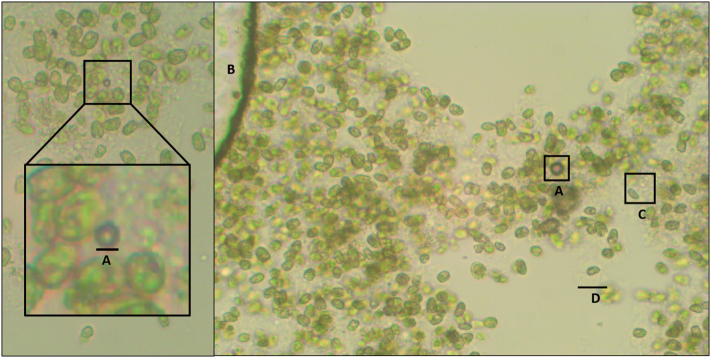
Surface foam from *T. chuii* cells embedded in a hydrogel matrix, spread onto a microscope slide and visualised at 400-fold magnification, following 30 s native electro-coagulation floatation (ECF) treatment in 1 L culture. (A) Microbubbles (smaller than 100 μm) can be seen embedded within the flocs in these images, however (B) much larger regions of trapped gas can also be observed, which continue to expand whilst being visualised under the microscope. (C) Cell debris can be seen within the image as small green clumps filling the space between cells, although there are also a high proportion of intact cells also present without obvious physical deformities following ECF treatment. (D) Halos of flocculant can be seen around cells in some cases, although it can be challenging to see as it is mostly transparent.

To further analyse the effect the flocculant was having on the culture and other particulates in solution, samples were taken for analysis with light microscopy and high speed atomic force microscopy (HS-AFM). Under light microscopy, the hydrogel appears transparent and is only visible because of the particulates embedded in it. This can be seen in Fig. 9, where the cells, gas bubbles and debris can be observed easily, but the hydrogel binding them together cannot. In this image, the motile species *T. chuii* is imaged embedded in the flocculant. Flagella could be seen moving on cells partially embedded in hydrogel, whilst the rest of the cell was anchored within the floc, resulting in the cells remaining stationary. Cells that were completely embedded within the flocculant showed no flagellar movement, showing how motile cells became trapped within the flocs. Visual inspection also highlighted that many cells in the image were still intact and had not been lysed through ECF treatment, although techniques to accurately determine survival rates and the concentrating effects were difficult to perform under flocculating conditions, as the biomass of the sample is concentrated within an unknown volume of floc along with cell debris and other detritus from the media, dry mass doesn't distinguish live-dead cells, the hydrogel changes the turbidity of the liquid, and reduces the permeability of stains, whilst also making individual counting techniques infeasible by gluing cells together.

As mentioned previously, some smaller microbubbles were visible embedded in the floc during light microscopy (Fig. 9). Whilst being observed, some of these grew rapidly, with the diameter more than tripling in some cases within 60 s of observation. This expansion is too large to be caused by the heat of the microscope lamp expanding the gas within the bubbles alone, and so must be combined with diffusion of other gases in solution, such as residual hydrogen from electrolysis or oxygen produced by photosynthesis. This also raises the question of whether the bubbles of a 1–10 μm scale are produced and trapped during ECF, or whether they form spontaneously as a side-product of photosynthesis/gas exchange after the cells are trapped within the hydrogel.

**Fig. 10:**
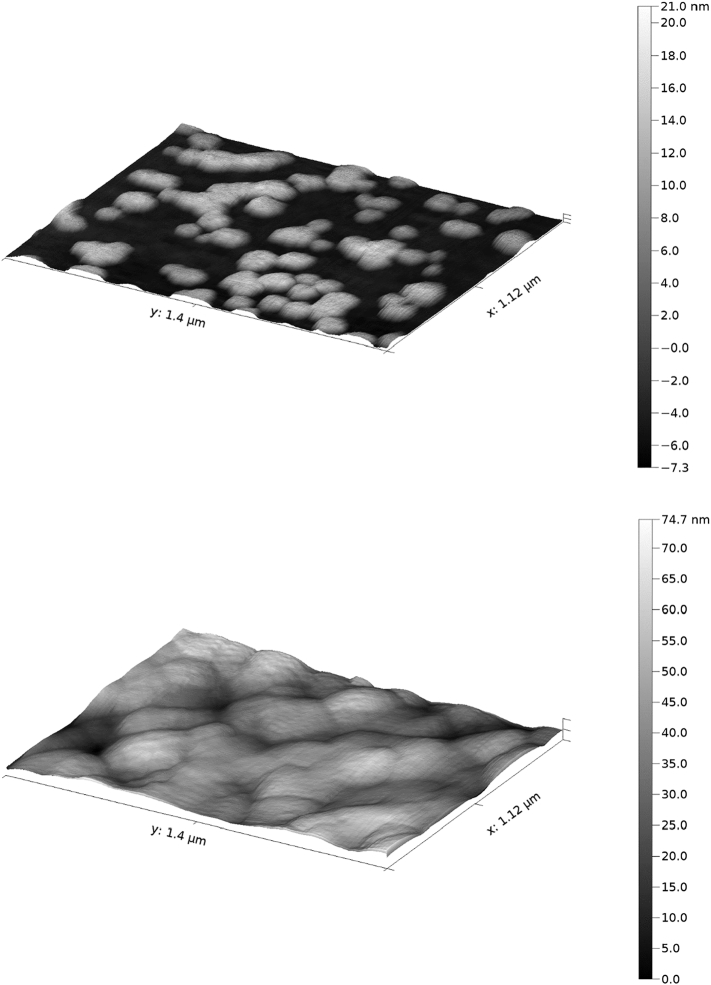
A single frame HS-AFM image of the hydrogel formed from ECF of blank media, where the material was spread thinly across a sheet of freshly cleaved mica and air dried. (*top*) At the sparsest level, very small individual spheroids can be seen, the smallest of which are approximately 40 × 80 × 20 nm in size. Larger individual spheroids can also be seen, as well as composite groups consisting of several merged together, forming the basis for the homogenous hydrogel. (*bottom*) At a higher concentration, with a continuous surface of hydrogel, the merged-spheroid model of floc surface is still present, with larger spheroids with a top surface of approximately 400 × 400 nm although the size range can vary. In this case, depth cannot be assessed as the flat mice surface is not visible in the image. The rippled effect that can be seen on the surface is an artefact of the imaging technique.

By itself, the flocculant appears as a feathery white solid when dried, and as a whitish hydrogel when wet (Fig. 1). When investigated by atomic force microscopy, the floc appeared to form nanometre scale clumps, which are generally spheroid when observed in isolation, that aggregate together. The smallest observable flattened spheroids were approximately 40 × 80 × 20 nm in size, although these also range in flat area up to 400 × 400 nm in size when forming part of a cohesive layer of hydrogel (Fig. 10). This shape is unlikely to be a result of how the samples were prepared on the mica, although arguably surface roughness may have been lost during evaporation, as areas with high surface area would lose water faster. Despite this, these structures are likely to be an accurate reflection of how the flocs seed within solution. The size difference in these two samples, which were taken from the same experiment, may be related to the level of evaporation that has taken place, where the denser gel has a larger reservoir of water trapped within it to reduce surface shrinkage, and so the larger spheroids may be a more accurate representation of initial floc seeding. The hydrogel has a nanoscale ‘bobbled’ surface, with a maximal roughness distance of 75 nm, approximate to an ISO grade roughness of N2 (average deviation <50 nm). This surface is also extremely sticky and caused repeated damage to the HS-AFM tips used during analysis.

**Fig. 11:**
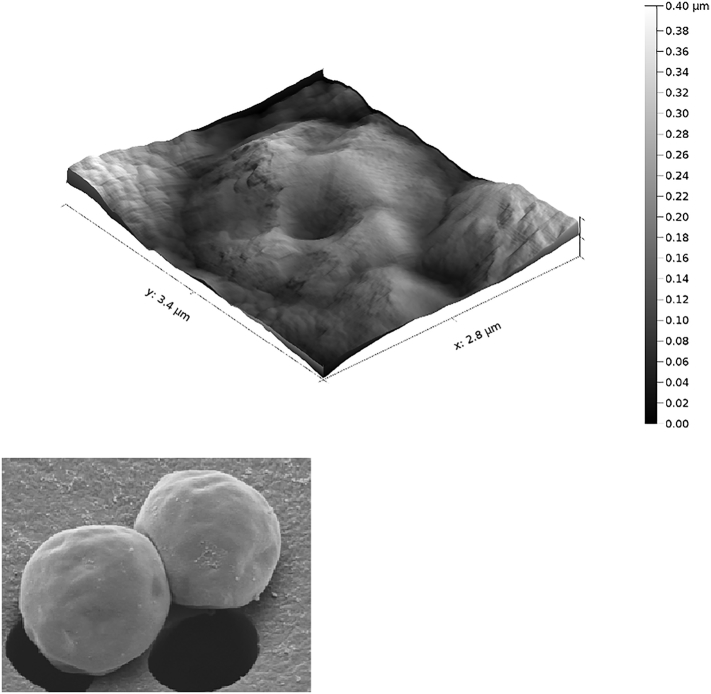
(Top) A single frame high-speed atomic force microscopy (HS-AFM) image of *C. sorokiniana* embedded within an electro-coagulation floatation (ECF)-derived hydrogel matrix, after 6 h of air-drying, where the material was spread thinly across a sheet of freshly cleaved mica and air dried. The reference scanning electron microscopy (SEM) image of the same *C. sorokiniana* strain (bottom) was kindly provided by S. Gschmeissner.

An AFM image of *C. sorokiniana* treated with ECF was taken to compare the changes that occur to the hydrogel when algae are present in the culture, and to assess how the hydrogel interacted with the surface of the cell (Fig. 11). The average spheroid size visible in dense floc ranges from 100–150 × 100–150 nm. This is smaller than the size observed in dense floc from ECF-treated media without cells, which may result from more of the Al(OH)_3_ adhering to cells and cellular debris as seed points, leaving a lower concentration to form isolated clumps in solution. In the centre of this image is a spherical cell, with 2.6 × 2.5 μm area and a 0.4 μm depth visible. For comparison, an SEM image of the same algal species is also included in the figure. Layers of the flocculant appear to coat the cell, creating a snowdrift-like effect of distinct layers or shells of hydrogel on the cell surface. This image shows that the cell membrane has developed pits, likely resulting from the air-drying preparation method. It is notable that there is much more pronounced roughness present on the algal floc than in the hydrogel layer, with a 400 nm maximal roughness in a microalgal containing sample (Fig. 10), compared with 75 nm in the floc-only sample (Fig. 11), suggesting that the presence of algae is a major contributing roughness factor in this image.

### 3.8 Removing the metal flocculant

For applications in which the biomass separated is to be processed into downstream products, the presence of the flocculant introduces unnecessary waste-mass and volume into the collected biomass that may need to be removed and possibly recycled, and where the product is to be introduced to farmland this has toxic potential, particularly in countries exposed to drought conditions. After centrifugation, a 1 L sample of *C. sorokiniana* grown to a density of ~500 mg L^−1^, with 10 g added NaCl, has a wet pellet size of approximately 5 mL. The same sample, treated with ECF for 90 s at 10 A, then treated with rapid mixing without ECF to disrupt all foam from the surface, has an additional dry mass of approximately 250 mg; and a wet pellet size of approximately 10 mL – equating to an approximate doubling of wet mass (5.0 ± 0.8 g vs 10.8 ± 0.3 g) due to additional retained media. Acidification of the hydrogel causes a chemical change that converts Al(OH)_3_ to a salt that lacks the hydrogel forming properties (Eq. (3)), however acidic conditions are also extremely damaging to algal cells and so a buffered solution was used to prevent cell lysis during treatment.

(3) $${\mathrm{3H}}^{+}X^{-}+\mathrm{Al}{\left(\mathrm{OH}\right)}_{3}\to {\mathrm{AlX}}_{\left(3\right)}+{\mathrm{3H}}_{2}O.$$

Acidification of aluminium hydroxide with acid creates aluminium-base salt and water, where H^+^X^−^ is any given acid with a pH of 5.5 or lower. Other aluminium salts do not have the same hydrogel-forming properties of aluminium hydroxide, and so dissolve, liberating the bound particles and cells in the process.

**Fig. 12:**
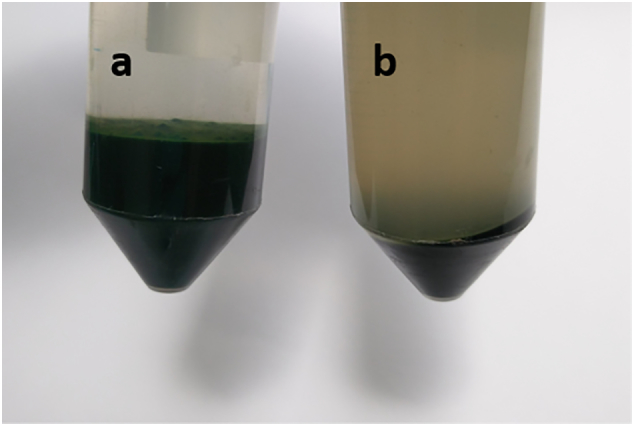
Image of the harvested cell pellets by electro-coagulation floatation (ECF), re-suspended and spun down with 20 mL of (a) water and (b) the oxalic acid buffer, respectively. The dark colour in the liquid in (b) is the resuspended metal salts, this colour became clear after 3 repeat washes, although a Palin test still showed an elevated level of soluble aluminium 100-fold higher than the water-only washed floc pellet.

An oxalic acid buffer solution [31] was used to dissolve the aluminium flocculant. The oxalate buffer was chosen from several different organic acid formulations, as it produced the least disruption to the infectivity of biological activity of marine viruses collected by metal flocculation, and so should theoretically be less disruptive to the surface chemistry of an algal cell. In addition, citric acid – a more obvious choice as an effective chelating agent, has been shown to form a human-toxic product when it neutralises Al(OH)_3_. A single treatment with 20 mL of this buffer on a 5–10 mL pellet collected from 1 L media, followed by three 35 mL distilled water washes, was sufficient to remove 85 ± 10% of the aluminium flocculant, as determined by ash analysis (Table 4). The free aluminium in solution was measured in the water washes by Palin-test, showing that aluminium released from the untreated pellet was 1.5 mg L^−1^ on the first water wash, and 0.08 mg L^−1^ after the 3rd wash, whilst the treated pellet was 190 mg L^−1^ on the first wash and 8 mg L^−1^ after the 3rd wash. The presence of the buffer disrupted the Palin-test readings on the initial buffer wash, and so accurate initial release values could not be measured (Fig. 12).

**Table 4:** Samples were weighed, calcinated at 450 °C for 2 h, then ash content was analysed. Al(OH)_3_ in the total ash weight was calculated by $\left(\mathrm{ECF}\;\mathrm{treated}\;\mathrm{ash}-\bar{\mathrm{ECF}\;\mathrm{untreated}\;\mathrm{ash}}\right)\times 1.5$ for each buffer wash condition, where 1.5 was the scalar to account for the 1/3 weight loss occurring in Al(OH)_3_ on heating above 180 °C.

ECF (s A)	Buffer	Pre-ash weight (mg)	Ash weight (mg)	Ash %	Al(OH)_3_ (mg)
900	Yes	50.47	5.14	10.18%	0.63
900	Yes	50.70	6.53	12.88%	2.72
900	Yes	50.30	5.85	11.63%	1.70
0	Yes	50.05	4.67	9.33%	0
0	Yes	50.17	5.17	10.30%	0
0	Yes	50.09	4.33	8.64%	0
900	No	50.29	13.02	25.89%	11.36
900	No	50.02	12.74	25.47%	10.94
900	No	49.99	12.62	25.25%	10.76
0	No	50.18	5.29	10.54%	0
0	No	50.07	5.03	10.05%	0
0	No	50.13	6.03	12.03%	0

Cells liberated with this buffer wash regime post-ECF, were found to be viable, however, determining accurate growth rates was challenging as residual hydrogel on the cells caused the cells to initially grow in concentrated clump in the centre of the shake-flask. Over time, the cultures became homogenous free floating cells, and passaging the cells from the liquid media of this sample at an equal concentration to those from an untreated control showed no differences in growth. Following confirmation that cells were viable, cells treated with ECF and harvested by centrifugation were compared with untreated cells captured by centrifugation, to determine if ECF had a negative effect on either lipid or pigment levels – two major economic foci of algal research.

Fatty acid methyl ester (FAME) profiles were generated for samples treated with and without ECF, and with and without subsequent oxalate buffer wash (Table 5). None of the treatments had any effect on the lipid compositions. Samples treated with ECF and subsequently buffer-washed showed no significant differences in total FAME per biomass level to unflocculated, buffer-washed cells (*p* = 0.709); however, samples treated with ECF that were not buffer washed did show a significant decrease in total FAME levels compared with an untreated control (*p* < 0.001). In addition, the buffer-washed ECF sample showed a significantly higher level of FAMEs than the unwashed ECF-treated sample (*p* = 0.024). FAMEs where generated by direct derivatisation of the dry biomass, and this observation suggests that aluminium hydroxide flocculant hydrogel provides some degree of protection for the cell wall against the high temperature, strong acid treatment that the cells are exposed to during the transesterification reaction either by reducing the efficiency of the reaction due to the neutralising chemistry of the aluminium hydroxide and/or by inhibiting cell lysis via the protective coating of the hydrogel. This effect appears to be concentration dependent as the residual hydrogel in the buffer-washed cells provided no additional protection against extraction. In addition, cells that were treated with the oxalic acid buffer appeared to have enhanced FAME levels compared with untreated cells.

**Table 5:** The summary data of fatty acid methyl ester (FAME) analysis, showing the conditions that were compared, the total proportion of FAMEs per mg biomass, and the saturated fatty acid (SFA), mono unsaturated fatty acids (MUFA) and poly-unsaturated fatty acids (PUFA). FAME given per mg biomass (adjusted for ash content).

Condition	μg FAME/mg biomass	SFA	MUFA	PUFA
No ECF-buffer	201.8 ± 4.3	11.1	7.1	81.8
No ECF + buffer	188.9 ± 15.5	11.8	6.8	81.4
ECF-buffer	156.6 ± 5.2	11.3	6.7	82
ECF + buffer	198.7 ± 15.4	11.3	8.5	80.2

**Fig. 13:**
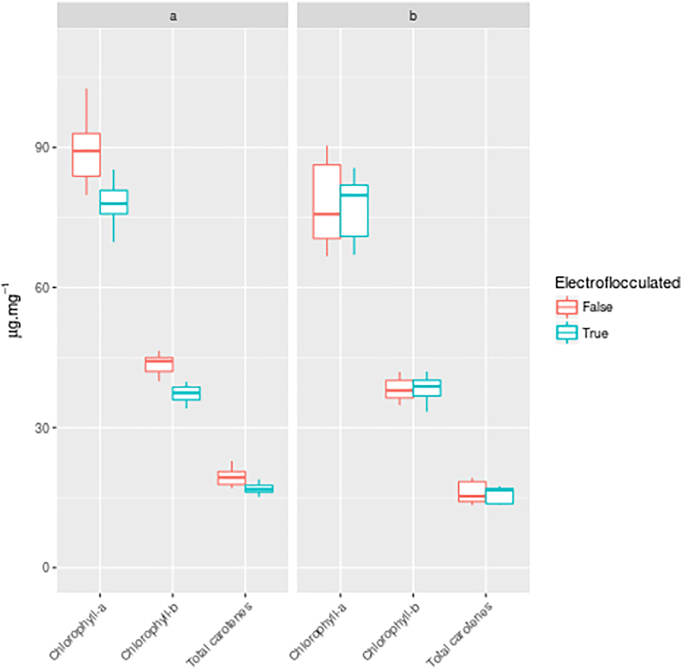
Spectrophotometric analysis of buffer-washed cells for chlorophyll-*a*, chlorophyll-*b*, and total carotenes in the samples treated with electro-coagulation floatation (ECF) (blue) against untreated samples (red). In the initial analysis (a) there was a significant difference in levels of detected pigments, however, when dried flocculant produced from ECF-treating blank media was spiked in at a 0.5 mg mg^−1^ (b) the significance was no longer observed and the variance in the observation values increased for all pigment measurements. (For interpretation of the references to colour in this figure legend, the reader is referred to the web version of this article.)

Chlorophyll a, b, and total carotenoid pigment levels were analysed by spectrophotometer. Samples treated with ECF and subsequently buffer-washed showed a significant, uniform reduction in observable pigment levels compared to untreated, buffer-washed cells (chlorophyll-a *p* = 0.00068, chlorophyll-b *p* = 1.718e-06, total carotenoids *p* = 0.002872), as seen in Fig. 13. It was noted that during cell disruption by bead beating for this analysis, the samples treated with ECF took far longer to attain a homogenous green colour than the untreated samples, suggesting a protective effect of the hydrogel against shear and mechanical disruption of the cells. To determine if the residual hydroxide was responsible for this, a repeat analysis was performed on the samples, where flocculant generated from ECF-treated blank media was spiked into all samples at a concentration of 0.5 mg mg^−1^ sample. Following this treatment, there was no longer an observable difference between the treated and untreated samples (chlorophyll-a *p* = 0.9261, chlorophyll-b *p* = 0.8237, total carotenoids *p* = 0.8219) (Fig. 13).

This suggests that the flocculant, even at low concentrations, can interfere with spectrophotometric analysis, as mentioned previously in attempts to measure growth rates or cell numbers after ECF treatment. This reduction may be a consequence of the floc causing small suspended particulates of cell debris containing unreleased pigment to drop out of solution, where they would normally contribute to the overall signal. It could also be that these particulates are still in suspension, but instead of being evenly distributed throughout the liquid being analysed, they are concentrated into micron-scale homogenous hydrogel spheroids. The large increase in the variance of the spectrophotometric measurements provides evidence for this latter scenario; however it is impossible to tell without further analysis exactly what is occurring at the micrometre and nanometre scale in solution. It is important to note that this observed reduction in signal post-flocculation may have important consequences for spectrophotometric analysis of other substances that have are exposed to even low levels of flocculating agent – a potentially important consideration for the water treatment industry, where flocculation is an important treatment step in wastewater processing.

### 3.9 Hydrothermal liquefaction (HTL)

**Fig. 14:**
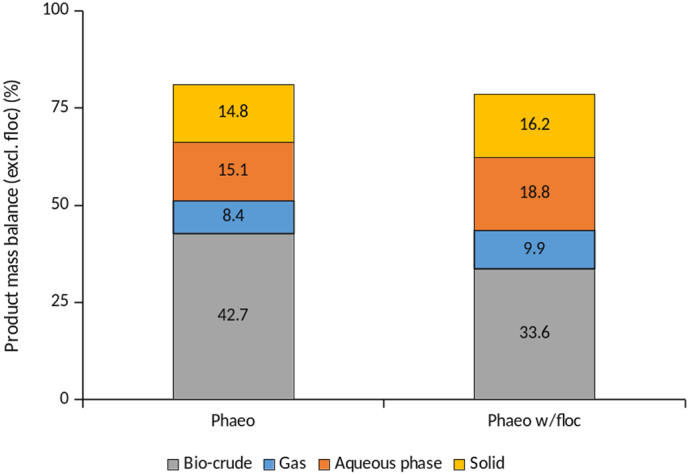
Relative proportions of different fraction following hydrothermal liquefaction (HTL) for *P. tricornutum* prior to, and following electro-coagulation floatation (ECF).

One potential application for flocculated algal biomass is the production of algal biofuel, through the hydrothermal liquefaction (HTL) of the biomass. The HTL process converts wet biomass (5–20% solids loading) into a bio-crude, gas, an aqueous phase, and a solid phase [35]. Excitingly, the algal biomass content in the flocculant was found to be 13.4% solids content, with 3.35% flocculant and 10.05% being algal biomass, ideal for direct hydrothermal processing. All HTL product yields were calculated relative to the flocculant-free mass of feedstock and presented on a DAF basis (the ash content of the algae was 12.4% of the dry algal biomass). During the HTL, a reduction in temperature was observed at around 200 °C, possibly due to the endothermic thermal decomposition of Al(OH)_3_ [38]. The inclusion of flocculant in the biomass feedstock led to a slight decrease in the overall production of bio-crude, at the expense of distributing carbon predominantly into the aqueous phase. A modest increase was also observed for the gas phase yield, suggesting that the presence of alumina promotes the formation of CO_2_ as well as other lighter organic species (Fig. 14). Al(OH)_3_ acts as a smoke-suppressant agent in polymers by promoting char formation through cross-linking, particularly in the presence of phosphate; which in this case may be responsible for more of the biomass content being found within the solid residue fraction [39].

## 4 Conclusions

ECF has significant potential as either a standalone separation protocol, or to add value to a pre-existing processing chain that uses centrifugation or some other gravity-driven separation. However, whilst the ECF technique is used widely in the treatment of a variety of wastewater sources, it is not currently in major use in the high value biotechnology sector despite previous work predicting processing costs to be as little as $0.19 per kg of microalgal biomass [28]. The main limiting factor to exploitation appears to be reservations about the contaminating aluminium hydroxide, which has been shown to interfere with downstream processing – such as in the case of HTL – or other analytical tests like growth rate, lipid, and pigment analysis. In addition, whilst aluminium is used in a variety of processes, including the food and pharmaceutical industry, there is known human toxicity associated with the metal – it is both neuro- and nephro-toxic [40,41], although in the Al(OH)_3_ form the aluminium is not absorbed by the human body [42], and is used in combination with magnesium hydroxide as an acidity regulator to treat acid reflux. The safety of Al(OH)_3_has been reviewed extensively elsewhere, where the major risk factor highlighted is that it is co-toxic in the presence of citric acid [40,41]. Despite these limitations, the Al(OH)_3_ provided support and protection to the cells against shear and acidic conditions, two conditions which are commonly encountered during downstream processing, and so ECF may have utility for protecting less robust cells in other biotech processes that might otherwise be challenging to harvest. Here, a buffer wash regime has been proposed that removes the Al(OH)_3_ from solution. Whilst the process still needs further refinement, the ability to remove the contaminating Al(OH)_3_ significantly improves the value of this technique in medium to large scale biotech industrial processing as an aid to volume reduction and target recovery.

Hydrogen gas is a by-product produced during the ECF process. With the use of aluminium electrodes, the rate of hydrogen production was found to be higher than expected from electrolysis, compared with other electrodes such as steel, further improving the economics of the process [43]. This hydrogen gas can be recycled to recover energy from the process, with studies showing ~54% of the total process energy being recovered in a batch process for nitrate removal performed at the 1 L scale [44], and up to 13% of the process energy recovered in a continuous flow system for blue dye removal with a working volume of 4.42 L [45]. In addition, the electrolysis process is driven by direct current, which makes it suitable to be driven from both photovoltaic panels and stored battery power, which has potential environmental benefits and displays the sustainable credentials of this technique. The inclusion of 1% NaCl prior to ECF of freshwater strains, whilst providing a useful opportunity for many established industrial microalgal platforms, is not without drawbacks: the creation of a brackish effluent may require additional treatment prior to discharge, and could thus incur additional costs. However, the benefits are legion in applications for smaller scale, higher value processes where for example cells or biologics are being generated for nutraceutical, pharmaceutical or cosmeceutical use, rather than the larger scale remediation applications to which ECF is currently applied. Indeed, the Al(OH)_3_ foam was found to be unstable for supporting gas within a foam in its native form, acting as a limiting step in the floatation aspect of ECF. Here, a casein additive in the form of powdered skimmed milk was investigated, which in combination with the Al(OH)_3_ created a more stable foam capable of capturing a much higher proportion of the gas produced during the process, improving separation and harvesting efficiency where floatation and surface scraping is used. This can be obtained as an additive cheaply from the dairy industry, with Alibaba listing the price range as $2500 - $3500 per ton at the time of writing – approximately $0.30 per 1000 L excluding additional process costs.

Substituting out the electrode material from aluminium to inert titanium oxide resulted in the in-situ production of hypochlorous acid, which has the potential to sterilise wastewater in a continuous flow regime. Coupled with online sensors, electrolysis could be used in cases where GM strains have been used to sterilise wastewater as it is evacuated from a large-scale process, or to sterilise a reactor that has become contaminated in-situ. As this process is electrically driven, it allows a machine-controlled system to limit dosing with feedback, without the need to have concentrated bleach stocks. At lower concentrations it also has the potential to sterilise media to drinking water levels without opening the reactor to contamination, at a concentration where the residual chlorine can be removed through air-bubbling alone. In addition, the first HS-AFM images taken of algal cells embedded in Al(OH)_3_ hydrogel in contact mode are presented here, showing the surface roughness of the flocculant and an algal cell embedded in the hydrogel on a nanometre level of detail, and an estimation of the microbubble range and concentration produced in solution at peak production. These details may be useful for future kinetic models for determining floc separation, without the need to revert to an experimental jar test. In summary, ECF continues to show great promise for exploitation in industrial processes for the removal, remediation or dewatering of microalgal suspensions. Here, two potential drawbacks hindering its application in higher value applications, namely the association of Al(OH)3 with biomass and the stability of the foam have at least partially been negated by the application of an oxalate buffer and powdered milk additive, respectively.

The following is the supplementary data related to this article.

Table S1 Media constituents of BBM with 1% NaCl added. **10 mg NaCl added to the media prior to ECF to increase energy efficiency of the process. Table S1

## Declarations of interest

None.
